# Effects of the Six Pillars of Lifestyle Medicine on the Performance of High School Athletes: A Systematic Review

**DOI:** 10.1177/15598276251388632

**Published:** 2025-10-14

**Authors:** Laeticia Evang, Joshua E. Lewis, Ernst J. Nicanord

**Affiliations:** 1 John Sealy School of Medicine, University of Texas Medical Branch, Galveston, TX, USA (LE, JEL); 2 Department of Family Medicine, University of Texas Medical Branch John Sealy Hospital, Galveston, TX, USA (EJN)

**Keywords:** lifestyle medicine, high school athletes, performance, adolescent health, well-being

## Abstract

Introduction: High school athletes face unique challenges, including balancing academic and athletic demands, addressing mental health concerns, maintaining adequate nutrition and sleep, and mitigating risks such as substance use and maladaptive behaviors. Female athletes frequently encounter additional issues, including amenorrhea and irregular menses. Lifestyle Medicine, encompassing nutrition, sleep, stress management, physical activity, avoidance of risky substances, and social connection, offers a promising framework for addressing these challenges through targeted, evidence-based interventions. Methods: A review of peer-reviewed studies from the last 15 years was conducted using databases like PubMed, Scopus, and Embase. Studies focused on athletes aged 14-18 were included, with 1423 studies screened and 31 meeting inclusion criteria. Studies on nonathlete populations and adults were excluded. Results: Of the 31 included studies, 6 addressed nutrition, 3 physical activity, 5 sleep, 5 stress management, 7 avoidance of risky substances, and 5 social connection. Notably, 52.1% of female athletes were found to be at risk for low energy availability (LEA), and over 79% of athletes reported sleeping fewer than 8 h per night, below the recommended range of 8-10 h for adolescents. Stress management emerged as a critical gap, with 91% of athletes reporting sport-related stress, yet only 27% received professional support. High-contact sports were associated with increased risks of substance misuse, including lifetime opioid use rates as high as 46% in some cohorts. Conclusion: This review underscores the urgent need for targeted interventions such as nutrition education programs, sleep hygiene initiatives, and mindfulness-based stress management tailored to high school athletes. Addressing these gaps within the framework of Lifestyle Medicine can enhance both performance and long-term well-being. Future research should evaluate the effectiveness of these interventions and explore their clinical and developmental implications.

“Nutritional deficiencies can have more profound effects on adolescent athletes, potentially leading to stunted growth, decreased bone density, and impaired muscle development.”

## Introduction

**Figure 1.**
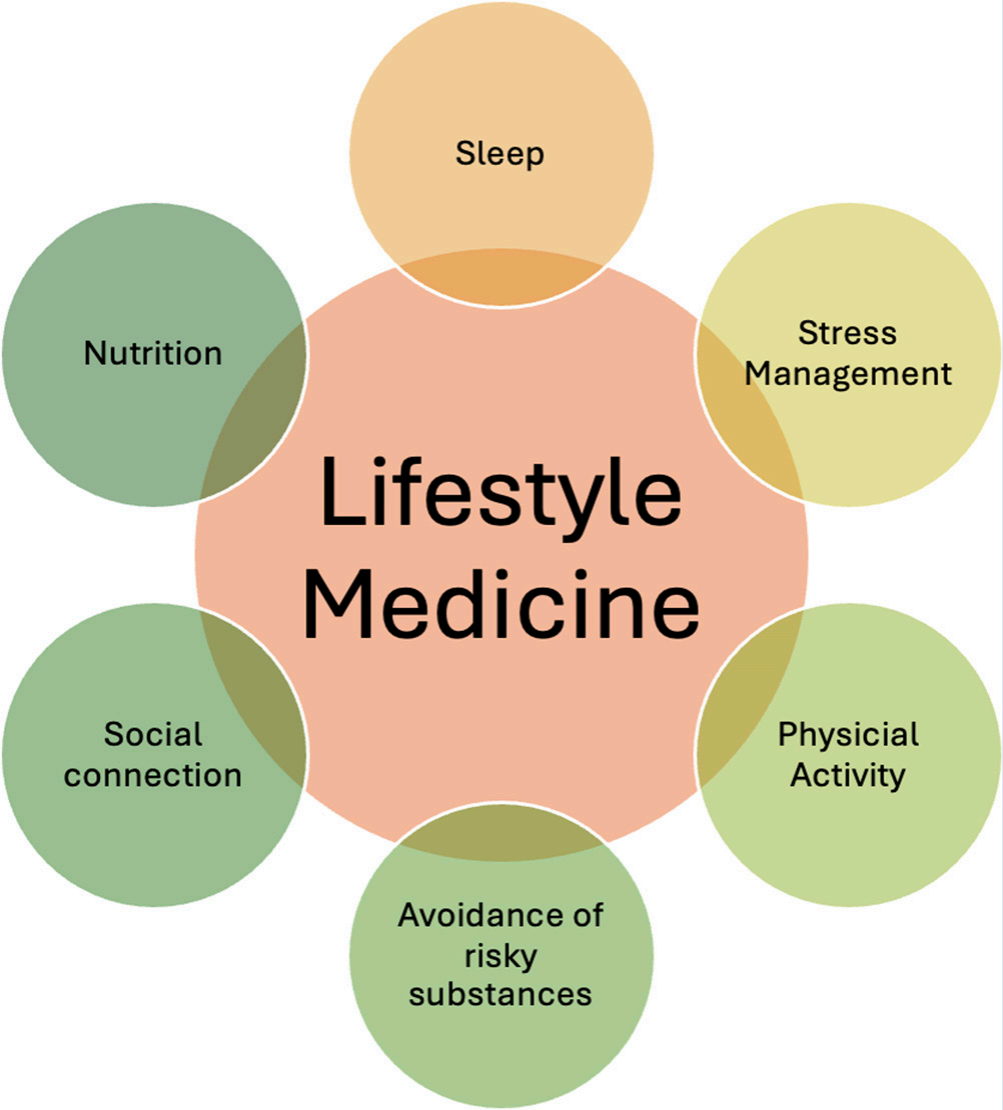
Six pillars of lifestyle medicine.

High school athletes face a unique set of challenges as they navigate the dual demands of academic and athletic excellence.^1^ Unlike professional or college athletes, this group is still in their formative years, making them particularly vulnerable to the effects of poor lifestyle choices.^2^ Many high school athletes train intensively and compete in high-stakes environments, often without the necessary support or knowledge to maintain their physical and mental well-being.^2^ The pressures to perform at a high level, coupled with academic responsibilities, create a perfect storm for burnout, injury, and diminished performance.^3^ It is in this context that Lifestyle Medicine, with its six pillars, nutrition, sleep, stress management, physical activity, avoidance of risky substances, and social connection, presents a powerful framework for optimizing health and performance in this demographic (Figure 1).

Adolescent athletes are not simply “smaller adults,” they undergo rapid physiological and neurodevelopmental changes that necessitate tailored health interventions.^4^ Puberty-related hormonal fluctuations, incomplete musculoskeletal development, and evolving neurocognitive systems make adolescents more susceptible to the effects of sleep deprivation, nutritional deficiencies, and unmanaged stress.^5,6^ For example, inadequate calcium or protein intake during adolescence may impair bone mineralization and muscle development, leading to higher injury risk.^7^ Similarly, the immature stress response systems of adolescents may amplify the psychological toll of competition, increasing their vulnerability to anxiety, depression, and burnout.^8,9^

Compounding these physiological vulnerabilities are systemic barriers that uniquely disadvantage high school athletes. Unlike collegiate or professional athletes who often have access to nutritionists, athletic trainers, and sports psychologists, high school athletes, especially those in under-resourced communities, may lack consistent access to these essential supports.^10,11^ Mental health services in school settings remain underfunded and understaffed, and lifestyle education is often not integrated into athletic programs. These limitations highlight the urgent need for accessible, developmentally appropriate interventions tailored to the high school athlete population.^10^ These six pillars are not only crucial for maintaining overall health, but they can also directly enhance athletic performance. For instance, proper nutrition fuels better endurance and faster recovery, while adequate sleep supports cognitive function and physical recovery, both essential for success on the field.^12,13^ Stress management techniques, such as mindfulness, help athletes stay focused and reduce anxiety during competition.^14,15^

However, while these benefits are well-documented in adult athletes, particularly in professional or college sports, there is a significant gap in understanding how these lifestyle factors interact in high school athletes. This gap in the literature is particularly concerning given the rising participation rates in high school sports and the growing recognition of the long-term health implications of poor lifestyle choices during adolescence.^16,17^ High school athletes are at a critical juncture in their development, where interventions targeting nutrition, sleep, and mental health could yield significant, long-lasting benefits.^18,19^ Yet, the lack of comprehensive research on how these factors work together to impact both performance and well-being in this group hinders the development of targeted, holistic strategies. Addressing this gap is essential for creating evidence-based guidelines that can help young athletes excel not just in their sport, but in their overall health and development.

The aim of this review is to address this critical gap by (1) examining the existing research on how the six pillars of Lifestyle Medicine affect high school athlete performance, (2) highlighting the specific challenges and needs of adolescent athletes, and (3) proposing future research directions and practical interventions to support both athletic and holistic health outcomes in this vulnerable population. By doing so, we hope to contribute to the development of evidence-based strategies that can enhance the well-being and performance of high school athletes, ensuring they have the tools they need to thrive both on and off the field.

## Methods

### Data Source

This review synthesized data from peer-reviewed studies identified through academic databases, including PubMed, Scopus, and Embase. A combination of search terms was used, such as “high school athletes,” “Lifestyle Medicine,” “nutrition,” “sleep,” “stress management,” “physical activity,” “substance avoidance,” and “social connection.” To ensure a thorough and robust data collection process, only studies published within the last 15 years were considered. Studies were included if they investigated the six pillars of Lifestyle Medicine in relation to athletic performance, with an emphasis on high school athletes.

### Screening and Quality Assessment

**Figure 2.**
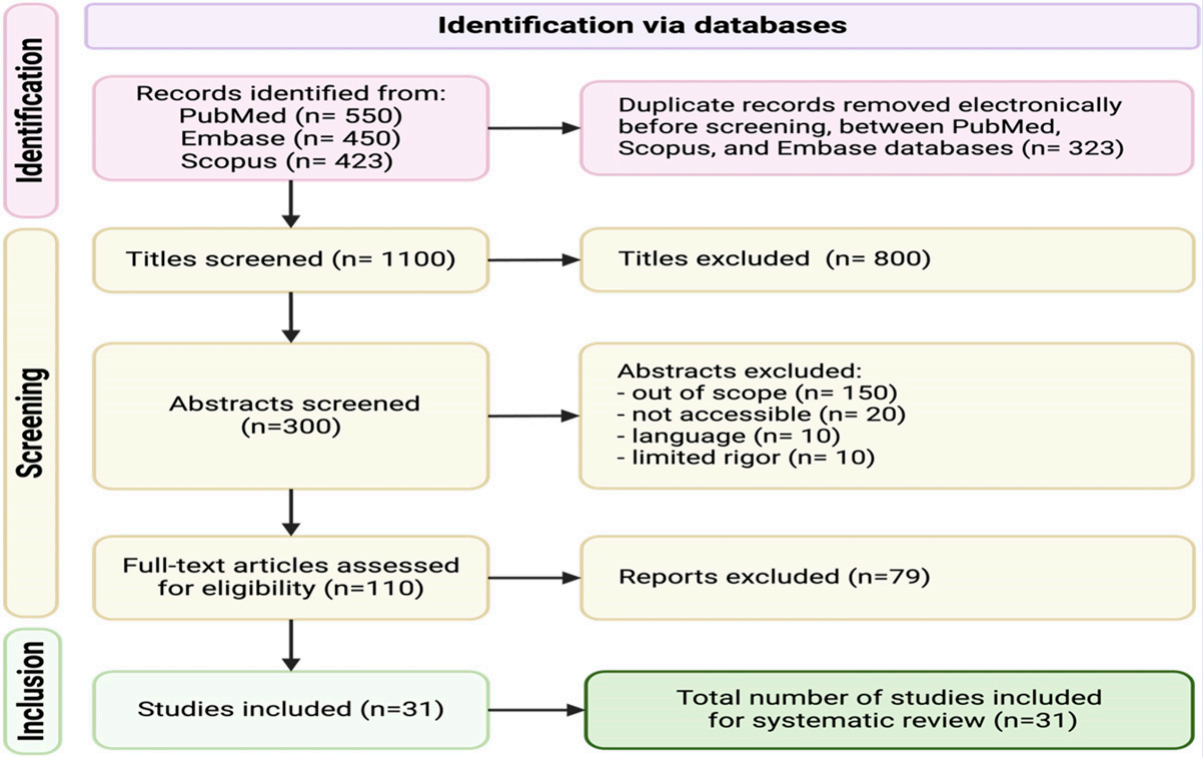
PRIMSA Flow Diagram: Flow diagram illustrating the identification, screening, eligibility assessment, and inclusion of studies for the systematic review. A total of 1423 records were identified through database searches. After removal of 323 duplicates, 1100 titles were screened. Of these, 800 were excluded, and 300 abstracts were reviewed. Following abstract screening, 190 were excluded based on criteria such as scope, accessibility, language, and methodological rigor. Full-text review was conducted for 110 articles, with 79 excluded for not meeting inclusion criteria. Ultimately, 31 studies were included in the final systematic review.

The screening process was conducted in 2 stages by 2 independent reviewers (J.L. and L.E.). Titles and abstracts were screened for relevance based on predefined inclusion and exclusion criteria. Discrepancies between reviewers were resolved through discussion, and if consensus could not be reached, a third reviewer (E.N.) was consulted for adjudication. No blinding was used during the review process. Full-text articles were then assessed for eligibility and methodological rigor. Although a formal scoring system such as the Newcastle-Ottawa Scale was not applied, studies were qualitatively assessed for clarity of research questions, appropriateness of study design, participant recruitment, outcome reporting, and overall transparency. Only studies demonstrating sufficient methodological rigor were included in the final analysis (Figure 2).

### Inclusion/Exclusion Criteria

The inclusion criteria for this review involved studies focusing on adolescent athletes aged 14-18 who participate in various sports, including football, basketball, soccer, track and field, and swimming. Priority was given to studies that directly investigated high school athletes’ lifestyle behaviors, specifically examining their alignment with the six pillars of Lifestyle Medicine. Only studies employing robust methodological designs, such as randomized controlled trials (RCTs), cohort studies, and cross-sectional studies, were included. Studies were required to demonstrate sufficient methodological rigor, including clear participant recruitment methods, appropriate controls, and transparent reporting of data and outcomes.

Exclusion criteria were applied to studies that focused solely on nonathlete populations or adult athletes, unless the findings were broadly applicable to adolescent athletes. Research on professional or elite athletes was excluded to avoid extrapolating results from populations with distinct training regimens and physiological responses. Additionally, studies not published in English or lacking rigorous study designs—such as poorly controlled or non-comparative designs, insufficient data transparency, or inadequate outcome measures—were excluded. This approach ensured that only high-quality, relevant studies were included in the final analysis.

### Variables

The key variables in this review are the six pillars of Lifestyle Medicine: nutrition, sleep, stress management, physical activity, avoidance of risky substances, and social connection. For each pillar, specific sub-variables were analyzed:

• **Nutrition**: Types of macronutrient intake (carbohydrates, proteins, fats), meal timing, and hydration strategies.^20-25^
• **Sleep**: Sleep duration, quality, and hygiene practices.^26-30^
• **Stress management**: Techniques used by athletes to manage competition-related anxiety, such as mindfulness, breathing exercises, and cognitive behavioral interventions.^1,3,15,31,32^
• **Physical activity**: Frequency, intensity, and variety of exercises, along with rest and recovery strategies.^33-35^
• **Substance avoidance**: Abstinence from alcohol, tobacco, performance-enhancing drugs, and other illicit substances.^36-42^
• **Social connection**: The presence and impact of support systems, including relationships with family, teammates, and coaches.^43-47^

### Outcomes

The outcomes measured in this review include a variety of factors related to athletic performance and well-being among high school athletes, specifically focusing on how each pillar of Lifestyle Medicine contributes to these outcomes. The review analyzed endurance, strength, agility, speed, and flexibility as key performance indicators, while cognitive outcomes such as reaction time, focus, and mental clarity were examined in relation to sleep and nutrition. Psychological well-being was assessed through stress levels, resilience, and burnout rates, with the added consideration of social support as a buffer against these stressors. Injury rates and recovery times from both acute and chronic injuries were also critical outcomes, highlighting the role of nutrition, stress management, and sleep in reducing injury risk and improving recovery. Additionally, the review explored long-term health outcomes, including the prevention of chronic conditions like substance use and social isolation, emphasizing their impact on an athlete’s long-term participation and overall well-being.

## Results

### Overview

The final results of this review include 31 studies that span a wide range of sports and lifestyle factors, with nutrition and physical activity being the most frequently studied variables. Specifically, the review found 6 studies on nutrition,^20-25^ 3 on physical activity,^33-35^ 5 on sleep,^26-30^ 5 on stress management,^1,3,15,31,32^ 7 on avoidance of risky substances,^36-42^ and 5 on social connection.^43-47^ This distribution reflects the multifaceted nature of lifestyle interventions relevant to adolescent athletes, with nutrition and substance avoidance emerging as the most frequently addressed areas. A total of 1423 records were initially identified across databases. After removal of duplicates and screening based on inclusion and exclusion criteria, 31 studies met the criteria for final inclusion. These studies encompass a diverse range of sports, most notably football, basketball, soccer, and track and field, and span geographic and demographic variations in adolescent athlete populations. A complete list of the studies by pillar and associated findings is included in Table 1. This synthesis offers a comprehensive understanding of how each lifestyle domain influences physical performance, psychological health, and long-term athletic development in high school athletes.

**Table 1.** Summary of Studies Included in Our Study.

Study	Origin	Study design and methods	Population	Patient sample size	Nutritional aspect investigated	Outcomes
Smith et al^20^	Not specified	General review of current knowledge, focusing on ethical limitations in youth nutrition research and adaptation of adult guidelines for youth	Young athletes	Not applicable (conceptual discussion)	Energy balance, training demands vs growth and maturation needs	Adult nutrition guidelines often repurposed for young athletes, but adjustments are needed to account for metabolic and physiological differences in youth; ethical considerations limit direct youth research
Petrie et al^21^	Not specified	General review of nutrient needs during exercise for sports in young athletes	Children and adolescent athletes	Not applicable	Fluid and electrolyte intake, energy requirements, carbohydrate intake, risk of nutrient deficits, micronutrient intake (iron, calcium)	Proper hydration and increased energy needs are critical; athletes in weight-control sports and female endurance athletes may be at risk of deficiencies in energy, protein, iron, and calcium. Inadequate nutrition may lead to acute (e.g., heat illness) and chronic issues (e.g., impaired growth, stress fractures)
Boisseau et al^22^	Not specified	Short-term repeated nitrogen balance study; participants consumed diets with varying protein levels (1.0, 1.2, 1.4 g/kg BW); nutrient and energy intake were tracked over 4-day periods during 3 weeks; nitrogen balance was assessed via urine analysis	Healthy 14-year-old male adolescent soccer players	11 participants (13.8 ± 0.1 years old)	Determination of daily protein requirements in adolescent athletes	The study found that protein requirements for adolescent male athletes are higher than the RDA for non-active adolescents, suggesting a need for 1.20 g/kg/day as the Estimated Average Requirement (EAR) and 1.40 g/kg/day as the Recommended Daily Allowance (RDA)
Manore et al^23^	Not specified	Cross-sectional survey study using 2 questionnaires (demographic/health history and sport nutrition)	High school soccer players, aged 14-18 years, from diverse backgrounds (55% female, 51% White, 41% Latino, 41% NSLP participants)	535 participants	Sport nutrition knowledge, behaviors, and beliefs, analyzed by sex, race/ethnicity, and socioeconomic status	Sport nutrition knowledge was 45.6% on average; disparities noted in NSLP-Whites vs NSLP-Latinos (higher in Whites, *P* < 0.01). Supplement knowledge lower in females (16%, *P* = 0.047) and Latinos (33%, *P* < 0.001). Breakfast consumption was lower among females and NSLP participants. Supplement use was higher in Latinos, with protein shakes more common among males and Latinos. 45% believed their nutrient needs differed from nonathletes. The study suggests a need for targeted nutrition education, especially for females and Latino athletes
Jeukendrup and Cronin^25^	Not specified	Review of existing research on metabolism and nutritional needs of young athletes, compared to adults	Children and adolescent athletes (elite)	Not applicable (review study)	Energy, carbohydrate, and fluid requirements; differences in metabolism between young and adult athletes	Young athletes differ from adults in energy metabolism, relying more on fat, with smaller glycogen stores and limited glycolytic capacity. They may require less carbohydrates but have a greater capacity to oxidize fat. Fluid and carbohydrate intake during exercise can benefit performance, though exact fluid requirements are unclear. Exogenous carbohydrate oxidation rates are similar between children and adults, due to absorption limits. More research is needed to develop age-specific nutritional guidelines for young athletes
Magee et al^24^	Not specified	Cross-sectional study using body composition assessments and electronic questionnaires (ASNK-Q, BEDA-Q, LEAF-Q for females only)	High school athletes, both male (n = 42) and female (n = 52); average age 18.09 ± 2.44 years	94 participants	Prevalence of low energy availability (LEA), risk of eating disorders, and sport nutrition knowledge	52.1% of female athletes were at risk for LEA. 42.9% of males and 68.6% of females were at risk for eating disorders, with higher risk among females (*P* < 0.01). Body fat percentage was inversely related to eating disorder risk. Sport nutrition knowledge was low across both sexes, with no significant difference between them. Female athletes with higher body fat percentage had a lower risk for eating disorders and LEA.
Escalona et al^35^	Spanish Elite Sport High School	Comparative study analyzing academic performance (GPA and University Entrance Examination scores)	Adolescents in the last Baccalaureate course, including elite athletes and control students (non-elite and recreational athletes)	1126 participants (570 girls, 18.2±0.6 years); 483 elite athletes, 643 controls	Not directly related to nutrition; focused on the relationship between sports participation and academic performance	Elite athletes had lower academic performance compared to control students (*P* < 0.001) across most subjects, regardless of sport type. The results suggest a need for programs that support the dual careers of young elite athletes, balancing both academics and sports
Carlton et al^34^	United States (North Carolina)	Observational study using direct observation (SOFIT) during 598 practice sessions across 12 high schools; regression model to analyze data	High school athletes participating in the 10 most popular boys’ and girls’ sports	Data collected from practices at 12 schools (exact participant number not specified)	Not directly related to nutrition; focused on physical activity levels during sports practices	Athletes engaged in moderate to vigorous physical activity (MVPA) 60% of practice time. MVPA levels varied by sport and were influenced by practice contexts. Sports like cross country and soccer (for girls) and cross country and track and field (for boys) had the highest MVPA. Practices that emphasized game simulation, fitness, and skill drills facilitated greater PA. Managing practice contexts can optimize PA to help athletes reach recommended activity levels
Bailey et al^33^	United States	Cross-sectional study using data from the 2019 United States Youth Risk Behavior Survey; multivariate regression models and t tests used for analysis	Adolescents aged around 15.94 years who experienced concussions and those who did not	1754 adolescents with a concussion history, 9795 without	Not directly related to nutrition; focused on mental health and risk behaviors following concussions	Adolescents with a history of concussions were more likely to report physical fighting, weapon carrying, and feelings of hopelessness. Male adolescents with concussions had an increased risk of suicide attempts. The study emphasizes the need for enhanced mental health support and safety measures for student-athletes and other at-risk youth
Ungaro and De Chavez^26^	United States	Objective sleep and physical activity data collected using Fitbit activity trackers over 8-14 consecutive days; analyzed sleep time, efficiency, bedtimes, wake times, and step counts	High school students (athletes and nonathletes), ages 15.8 ± 0.8 years and 16.3 ± 0.7 years, respectively	34 student-athletes (18 males, 16 females) and 38 nonathletes (10 males, 28 females)	Not directly related to nutrition; focused on sleep and physical activity	Student-athletes and nonathletes did not differ significantly in total sleep time or efficiency. Most participants did not meet the recommended 8 h of sleep. Student-athletes took more steps daily. Earlier bedtimes correlated with longer sleep duration, and earlier wake times correlated with more steps. Consistent, earlier sleep/wake schedules may lead to healthier outcomes
Potter et al^27^	United States	Cross-sectional study; participants completed the Pittsburgh Sleep Quality Index (PSQI) and the PROMIS Pediatric Profile 25 questionnaire; analysis adjusted for sex and age	Uninjured high school athletes, mean age 15.3 ± 1.1 for poor sleepers, 15.1 ± 1.7 for good sleepers	110 athletes with poor sleep quality (62% female), 162 athletes with good sleep quality (33% female)	Not directly related to nutrition; focused on sleep quality and its impact on QOL	Poor sleep quality was associated with worse ratings for physical function, anxiety, depressive symptoms, fatigue, pain interference, and pain intensity. Clinicians should address sleep hygiene to improve QOL in adolescent athletes
Sufrinko et al^28^	Not specified	- Baseline ImPACT and PCSS assessments	Athletes aged 14-17 years (M = 15.26, SD = 1.09)	7150 athletes, with 78 symptomatic sleep-restricted adolescents and 99 asymptomatic optimal sleepers for follow-up analysis	Not applicable (the study focused on sleep duration and symptoms rather than nutritional aspects)	- Dose–response effect of sleep duration on neurocognitive performance was significant (Wilk’s λ = .98, F (27 145) = 17.25, *P* < .001, η^2^ = .01)
						- Poorer performance in symptomatic sleep-restricted adolescents on cognitive measures:
		- Participants divided into 3 sleep duration groups:				- Verbal memory (F = 11.60, *P* = .001)
		1. Sleep restriction (≤5 h)				- Visual memory (F = 6.57, *P* = .01)
		2. Typical sleep (5.5-8.5 h)				- Visual-motor speed (F = 6.19, *P* = .01)
		3. Optimal sleep (≥9 h)				- Reaction time (RT) (F = 5.21, *P* = .02)
		- MANCOVA and follow-up MANOVA analysis				- Girls in the sleep problem group performed worse on RT (*P* = .024)
Moran and Ingargiola^29^	Not specified	- Retrospective analysis of ImPACT performance	High school athletes aged 13-19 years	958 athletes	Not applicable (the study focused on sleep quantity rather than nutritional aspects)	- Significant differences in baseline symptom factors and total symptom scores:
		- Participants categorized into 2 groups based on self-reported sleep quantity:				
		1. <8 h (n = 524; 55%)				- Greater symptoms reported in the <8 h sleep group (ps < .02 for symptom factors, *P* < .001 for total symptom scores)
		2. ≥8 h (n = 434; 45%)				- No significant group differences in composite scores for verbal memory (*P* = .49), visual memory (*P* = .94), visual-motor speed (*P* = .38), reaction time (*P* = .50), or impulse control (*P* = .81)
		- Measures included baseline total symptom scores and symptom factors (vestibular-somatic, sleep-arousal, affective, cognitive-sensory) as well as ImPACT composite scores (verbal and visual memory, visual-motor speed, reaction time, impulse control)				- Conclusion: High school athletes with <8 h of sleep reported greater symptoms, indicating clinicians should consider sleep adequacy for valid assessments
Duffield et al^30^	Not specified	Retrospective cohort study; measurements included depressive symptoms (PHQ-9), anxiety symptoms (GAD-7), somatic complaints (PHQ-15), and sleep quality (PSQI) using cutoff scores of ≥6 and ≥8 to indicate poor sleep quality	Collegiate athletes (male and female) across 5 collision, contact, and limited contact team sports	137 athletes	Not applicable (the study focused on sleep quality and its predictors rather than nutritional aspects)	- **Poor sleep quality**: 53% of athletes with PSQI ≥6 and 33.5% with PSQI ≥8
						- **Predictors of poor sleep**: Depressive symptoms, somatic complaints, Caucasian race, male sex, and number of concussions were significant predictors (*P* < .05)
						- The model explained 43% of the variance in PSQI, with depressive symptoms accounting for 9% of the variability
						- Anxiety symptoms, sport category, and history of migraines were not significant predictors of poor sleep quality
Ward et al^31^	United States	Cross-sectional survey; anonymous online survey platform assessing stress, coping, and desire for professional help	High school athletes aged 16-17; both male and female from various sports, locations, and ethnic backgrounds	200	Not directly related to nutrition; focused on stress and coping in sports	91% reported experiencing stress due to sports; 27% of those with moderate to extreme stress wanted help but did not receive it. Only 18% thought professional help would be unbeneficial. Common stress sources included fear of failure and self-pressure. Stress may be linked to future anxiety and depression. Access to medical support is essential
Petterson and Olson^48^	Various	1 randomized control trial (RCT), 2 non-randomized control cohort studies; assessed the effects of mindfulness-based interventions on stress, injury reduction, and quality of life	Student-athletes aged 13-24 years	3 studies with varying sample sizes	Indirectly related to nutrition; focused on stress management, injury prevention, and overall well-being through mindfulness	Improved levels of mindfulness, reduced perceived stress, and negative thoughts; decreased number of injury occurrences following intervention. Grade B evidence supports the use of mindfulness-based interventions for reducing stress, improving well-being, and potentially reducing injury risk due to enhanced mental awareness and acceptance
Wilczynskaet al^3^	Various	Meta-analysis of 5 studies (published between January and June 2022); assessed the effects of psychological interventions on athlete burnout following PRISMA guidelines and Cochrane risk of bias assessment	Young athletes	5 studies with varying sample sizes	Indirectly related to nutrition; focused on reducing burnout through psychological interventions	Cognitive behavioral therapy (CBT) and mindfulness-based interventions effectively reduced burnout dimensions; online interventions were particularly beneficial. Conclusions highlight the need for more high-quality studies on this topic due to the physical and psychological impacts of burnout on athletes
Lundgivst et al^1^	Sweden	Qualitative study using semi-structured interviews; thematic analysis performed on data collected from athletes	Elite-striving lean sports athletes aged 16-18 in sports high schools	8 participants (2 men, 6 women)	Investigated eating pathologies related to body weight demands in sports	Identified key themes: Integration for future career success, psychosocial stress (including body weight demands and eating disorders), protective psychosocial factors (social support, communication, self-care), and support needs for junior-to-senior transition (career advice, individualized support). Highlighted inadequate support in sports developmental programs, ongoing stigma around mental health, and the taboo nature of discussing eating disorders
Delfin et al^32^	Alabama, USA	Cross-sectional study using self-reported measures on the NIH Toolbox Application and PROMIS	Black and African American adolescent football athletes	93 participants	Examined the relationships among social support, psychological stress, and mental health symptoms (depression, anxiety)	Findings indicated that social support (emotional support, family, peer relationships) was negatively correlated with psychological stress and depression. Stress and mental health symptoms (depression, anxiety) were positively correlated. Social support predicted lower psychological stress and depression symptoms, suggesting a stress-buffering effect that may preserve mental health among adolescent athletes. Further research is needed, especially focusing on racial and ethnic minorities
Williams et al^41^	Canada	Cross-sectional study using data from the COMPASS study, 2017-2018	Male and female high school students	60 601 participants	Examined the association between school sports participation (none, intramurals, varsity, both) and substance use behaviors (binge drinking, cannabis, cigarette, and e-cigarette use)	Intramurals were protective against cannabis and cigarette use among all students and e-cigarette use among females. Varsity sports were protective against cannabis and cigarette use but associated with higher odds of binge drinking and e-cigarette use. Students participating in both sports types had lower odds of cannabis and cigarette use but higher odds of binge drinking and e-cigarette use. Findings suggest that substance use prevention should be a focus for varsity sports teams, especially concerning binge drinking and e-cigarettes
Naylor et al^40^	Massachusetts, USA	Survey-based cross-sectional study with 150-item survey assessing substance use	High school athletes and nonathletes	1515 students	Relationship between athletic participation, lifestyle habits, and recreational drug use	Athletes were significantly less likely to use cocaine, psychedelics, and smoke cigarettes compared to nonathletes. However, athletes were more likely to use creatine, with no difference in anabolic steroid and androstenedione use. The study suggests that athletes generally lead healthier lifestyles, but drug intervention programs may not be fully effective, requiring further evaluation
Veliz et al^39^	USA	Longitudinal, multicohort panel study using data from the Monitoring the Future Study (2006-2017)	High school seniors (12th-graders) followed into young adulthood	4772 participants	Association between involvement in high-contact, semicontact, or noncontact sports during 12th grade and initiation of prescription drug misuse (PDM)	Approximately 31% of high school seniors indicated PDM at baseline. Participation in noncontact and contact sports increased the odds of initiating prescription stimulant misuse over the following 10 years compared to nonathletes. This study suggests that sports participation in high school is associated with higher risks of prescription stimulant misuse into young adulthood, highlighting the need for PDM screening during adolescence and ongoing monitoring, especially among athletes
Johnson and McRee^38^	USA	Descriptive study using data from 2 statewide surveys: a survey of adolescents and a survey of nurse practitioners and physicians	High school athletes and healthcare providers	46 492 adolescents, 561 providers	Examined the prevalence of health-risk behaviors among high school athletes and the proportion of providers who address these behaviors during PPEs	The most prevalent risk behaviors among athletes were low levels of physical activity (70%), bullying perpetration (41%), and alcohol use (41%). Although most providers addressed common risk behaviors during PPEs, fewer addressed issues such as bullying, violence, and prescription drug use. Topics discussed differed by provider type and patient population, suggesting a disconnect between the issues athletes face and those addressed during PPEs
Forman et al^37^	USA	Cross-sectional study using survey data on high-risk behaviors	Adolescent male athletes (ages 13-19) and male and female nonathletes	Unknown	Prevalence of drug use and sexual activity in athletes compared to nonathletes	The study found a significantly lower prevalence of drug use (e.g., beer, cigarettes, marijuana) among male athletes in their senior year compared to nonathletes, suggesting sports participation may decrease substance use. However, a higher proportion of sexually active athletes had their first sexual intercourse between ages 13 and 15, compared to nonathletes, indicating potential earlier engagement in sexual activity. These trends persisted throughout high school. Findings suggest that while athletics may promote a decrease in drug and alcohol use, it may also lead to earlier initiation of sexual contact
Wetherill and Fromme^36^	Not specified	Cross-sectional study; web-based surveys assessing alcohol use, sexual activity, sports participation, and perceived risk; Generalized Linear Modeling (GLM) used for analysis	College-bound high school graduates	2247 participants	Influence of perceived invincibility on alcohol use and sexual activity in high school athletes vs nonathletes	Athletes reported greater alcohol use, more sexual partners, and lower perceived risk. Perceived risk mediated the association between sports participation and alcohol use, as well as sexual activity, particularly for women
Ekhtiari et al^42^	Various studies analyzed in a systematic review	Systematic review including survey studies and retrospective observational designs; data from Embase, MEDLINE, and PubMed	Athletes (high school, professional)	226 256 athletes across 11 studies	Rates of opioid use and misuse, differences compared to non-athletic populations, risk factors, and subgroup risks	Opioid use among professional athletes ranged from 4.4% to 4.7% at any given time, with a lifetime use of 52% in the NFL. High school athletes had lifetime opioid use rates of 28% to 46%. Risk factors included Caucasian race, contact sports, postretirement unemployment, and undiagnosed concussion. Opioid use during a playing career predicted postretirement use
Bates et al^43^	United States	Qualitative study; 7 focus groups conducted with diverse school districts	High school student-athletes	50 student-athletes	Experiences of student-athletes related to structural, contextual, and psychosocial factors influencing well-being	Student-athletes expressed a desire to excel post-COVID-19 restrictions but felt challenged by the demands. Emergent themes included academic pressures, sport specialization, and mental health concerns. Findings highlight the need for tailored interventions, coach training, and supportive school policies to address the holistic well-being of student-athletes
Murray et al^44^	Unspecified	Two studies: Study 1 - cross-sectional survey; Study 2 - longitudinal assessment over 6 months	Athletes	Not specified	Relationship between social support, social identification, and burnout	Higher levels of social identification in a sport strengthened the protective effect of perceived social support against burnout. Both studies found that social identification moderated the negative association between social support and burnout, suggesting that social identity enhances the benefits of social support in reducing burnout symptoms
Burke et al^45^	Unspecified	Systematic review of 12 studies (quantitative, qualitative, mixed-methods)	Youth sport parents	N/A (Review of existing studies)	Efficacy, design, and evaluation of parent-education programs targeting psychosocial parental support	All 5 quantitative studies showed significant improvements. Qualitative studies noted enhanced parent knowledge and skills. Only one mixed-methods study had significant results, but qualitative data suggested improved parent-athlete relationships. The review highlighted the lack of use of behavior change theories and absence of sport-specific measures for evaluating changes in parental behavior. Future programs should incorporate validated, theory-based approaches for better outcomes
Dorsch et al^46^	Unspecified	Qualitative study using semi-structured focus groups	Youth sport parents	26 parents across 5 focus groups	Parent socialization through children’s organized youth sports	63 themes identified, reflecting parental changes in behavior, cognition, affect, and relationships. Six moderators influencing parental socialization were noted: Child age, parent past sport experience, parent/child gender, child temperament, community sport context, and type of sport (individual vs team). Categories of parental change were found to be interconnected, indicating complex socialization processes. Future research should explore these dynamics further to improve understanding of parent behavior in youth sports
Williams and MacNamara^47^	Unspecified	Qualitative study using semi-structured interviews	Young athletes in the early stages of the talent pathway	8 participants	Experiences of young athletes as they navigated their most difficult challenges on the talent pathway	Participants highlighted the unique and significant impact of their challenges, the use of diverse psychobehavioural skills, and the role of social support in overcoming difficulties. These experiences influenced their preparation for future challenges. Stakeholders in talent development should focus on helping young athletes deploy relevant skills and provide support to navigate challenges effectively, emphasizing the need for individualization, preparation, and reflection processes

### Physical Activity

Physical activity plays a crucial role in the physical and cognitive development of high school athletes.^33-35^ Engaging in sports and organized physical activities not only helps students meet the recommended levels of moderate to vigorous physical activity (MVPA) but also contributes to their overall fitness and cognitive performance.^35^ Research shows that high school athletes accrue substantial amounts of MVPA during practice, with variations across different sports based on practice context and structure.^34^ Sports that prioritize game simulation, fitness, and skill development tend to promote higher levels of MVPA. However, while physical activity is associated with improved fitness and academic performance in non-elite athletes, elite athletes may face challenges in balancing their rigorous training schedules with academic demands, often resulting in lower academic performance compared to their non-athletic peers. However, participation in sports, particularly contact sports, heightens the risk of concussions, which can lead to both physical and mental health impairments, including increased risk of suicidality and hopelessness.^33^ These findings underscore the need for comprehensive support systems that address both the physical and academic needs of high school athletes, ensuring that they can pursue excellence in sports while safeguarding their mental health and academic success.

### Nutrition

Nutrition plays a fundamental role in the performance, development, and overall health of high school athletes.^20-25^ Adolescents engaged in high levels of physical activity require adequate and balanced nutritional intake to support not only their athletic performance but also their growth and maturation.^21^ Studies show that many young athletes, particularly females, are at significant risk for low energy availability (LEA), with over 52% of female athletes found to be at risk.^24^ This can have far-reaching consequences, including decreased performance, increased susceptibility to injuries, and delayed recovery. Moreover, inadequate intake of essential nutrients like carbohydrates and protein is particularly concerning for endurance athletes, who often experience deficits in calcium and iron, leading to issues such as stress fractures and impaired bone health.^20,21^ The link between nutritional deficiencies and injury risk underscores the critical need for focused nutritional interventions in this population. Additionally, research has found that adolescent athletes, particularly females and Latinos, often have limited sports nutrition knowledge, with females scoring 16% lower than males and Latinos scoring 33% lower than White athletes.^23^ This knowledge gap, combined with disparities in dietary habits—such as lower breakfast consumption in females (50%) compared to males (60%)—further highlights the need for targeted nutrition education to optimize both short-term performance and long-term health.^23^ Protein intake is another critical factor, particularly for male athletes. Studies have shown that the protein requirements for adolescent male athletes, such as 14-year-old soccer players, are significantly higher than the recommended daily allowance (RDA) for non-active adolescents.^22^ Protein needs in these athletes are estimated to be 1.40 g/kg/day due to the demands of physical training. Without proper nutrition, these athletes face not only short-term performance detriments but also long-term health risks, reinforcing the importance of developing evidence-based nutritional strategies for young athletes.

### Sex-Specific Findings in Female Athletes

Several studies in this review identified sex-specific vulnerabilities among female high school athletes, particularly related to nutrition and reproductive health. Magee et al reported a 52.1% prevalence of low energy availability (LEA) among female athletes, with over two-thirds (68.6%) of those at risk for disordered eating behaviors.^24^ LEA was significantly associated with menstrual irregularities, including delayed menarche, oligomenorrhea, and secondary amenorrhea, reflecting disruptions in the hypothalamic-pituitary-gonadal axis due to inadequate caloric intake. These reproductive health issues not only pose risks to short-term athletic performance but also carry long-term consequences for bone health and metabolic function. Additionally, the psychological stress linked to body image concerns and performance pressures may compound these risks, as inadequate stress management strategies were noted across several studies. These findings emphasize the urgent need for sex-specific screening tools and interventions tailored to the physiological and psychosocial needs of adolescent female athletes.

### Stress Management and Benefit of Social Support

Stress management is a critical aspect of maintaining the overall well-being and performance of high school athletes, particularly those participating in demanding sports such as football.^1,3,31,32,48^ A cross-sectional study of Black and African American adolescent football players in rural Alabama revealed that social support plays a significant role in buffering psychological stress and preserving mental health.^32^ Emotional and familial support were found to be negatively correlated with psychological stress (r = −0.386 and r = −0.412, respectively) and mental health outcomes like depression (r = −0.367 for emotional support, r = −0.323 for family relationships). Additionally, research highlights that mindfulness-based interventions are effective in reducing perceived stress and the number of injury occurrences in athletes.^31,48^ For example, a review of 3 studies demonstrated that mindfulness strategies significantly increased mental awareness and mitigated negative thoughts, leading to improved quality of life.^48^ Furthermore, approximately 91% of high school athletes surveyed across different sports reported experiencing stress due to athletic participation, with fear of failure and self-pressure as the most common stressors.^31^ While a third of athletes believed stress positively affected their performance, nearly 27% sought but did not receive professional help for their stress.^31^ The integration of both social support systems and mindfulness-based strategies may provide a comprehensive approach to stress management, helping athletes navigate the psychological challenges inherent in sports.

Social support plays a pivotal role in helping young athletes navigate the complex challenges of their athletic journey, especially in the early stages of their talent development.^43-47^ Research indicates that athletes facing their most difficult challenges can significantly benefit from a diverse support network, which not only aids in managing the immediate stress but also prepares them for future challenges.^47^ Psychobehavioral skills and key social support from coaches, family, and peers are essential in fostering resilience and adaptability during these times.^43,44,46^ Moreover, parent-education programs have been shown to enhance positive parental involvement, thereby reducing stress for both parents and youth athletes.^45,46^ These programs, though still underexplored, have demonstrated improvements in parental knowledge and the parent-athlete relationship, which in turn provides a more supportive environment for the athlete. Additionally, the perceived availability of social support has been linked to reduced symptoms of burnout, especially when athletes strongly identify with their sport.^44^ This underscores the importance of creating a strong sense of community and shared identity within teams, as it enhances the effectiveness of social support in mitigating burnout. High school athletes, particularly in the post-pandemic era, also face heightened psychosocial stressors related to academic pressures, sport specialization, and mental health concerns. These findings highlight the critical need for comprehensive social support systems to help athletes manage the increasing demands of both their sports and academic environments.

### Sleep and Sleep Management

Sleep duration and quality are critical components in assessing the overall health and performance of high school athletes.^26-30^ Research consistently shows that high school athletes often fail to meet the recommended 8-10 h of sleep per night, which is considered essential for good quality sleep. In one study, 79% of athletes and 87% of nonathletes slept less than 8 h per night (*P* = .82), falling short of the National Sleep Foundation’s guidelines for adolescents.^26^ Sleep quality, however, cannot be defined by duration alone. For instance, while both groups averaged approximately 7.3 h of sleep, the athletes had a slightly higher sleep efficiency (93.6% ± 2.3% vs 92.9% ± 2.3%, *P* = .20), indicating that quality does not solely depend on total sleep time but also on the body’s ability to sustain restful sleep throughout the night.^26^

Studies also demonstrate the tangible effects of inadequate sleep on athletes’ mental and physical health.^27,28^ Athletes reporting poor sleep quality were found to be more likely to experience fatigue (β = 0.537; *P* < .001), more likely to have depressive symptoms (β = 0.456; *P* < .001), and significantly more prone to pain interference (β = 0.247; *P* < .001) and pain intensity (β = 0.103; *P* = .006).^27^ Additionally, a dose–response effect has been observed where athletes sleeping fewer than 5 h exhibited poorer neurocognitive performance—significantly lower scores in verbal memory (F = 11.60; *P* = .001), visual memory (F = 6.57; *P* = .01), and reaction time (F = 5.21; *P* = .02)—compared to those getting optimal sleep (≥9 h).^28^ These findings underscore that both the quantity and quality of sleep have profound impacts on athletic and cognitive performance, emphasizing the need for sleep interventions to enhance overall well-being and performance in high school athletes.

### Avoidance of Risky Substances and Risky Behavior

Participation in high school sports offers significant physical and psychosocial benefits, but it also presents potential risks, particularly regarding substance use and risky behaviors.^36-42^ Studies suggest that while athletes tend to engage in fewer illicit drug activities than their nonathlete peers, there are concerning trends related to alcohol consumption and sexual activity.^37,38^ For instance, athletes in their senior year of high school were found to have lower rates of alcohol use (by 25.5%), cigarette smoking (by 57.5%), and marijuana use (by 57.7%) compared to nonathletes.^37^ However, they were more likely to engage in binge drinking and had a higher number of sexual partners, with perceived risk mediating the association between sports participation and alcohol use as well as sexual activity.^36,38^ Among male athletes, 45.5% reported never having had sexual intercourse compared to 50% of nonathletes, with 81.9% of sexually active athletes initiating sexual intercourse between ages 13 and 15, compared to 67.8% of nonathletes.^37^ Additionally, sports participation in 12th grade was associated with increased odds of prescription stimulant misuse over the following decade (adjusted OR = 1.57 for contact sports and OR = 1.40 for noncontact sports).^39^

Similarly, findings from the COMPASS study revealed that while intramural sports participation was protective against cannabis and cigarette use, varsity sports were associated with higher odds of binge drinking and e-cigarette use.^41^ Moreover, opioid use is another pressing concern in the athletic population, with high school athletes reporting lifetime opioid use rates between 28% and 46%, comparable to those of professional athletes.^42^ Risk factors for opioid misuse include participation in contact sports such as football and wrestling.^42^ These findings emphasize the need for targeted substance use prevention efforts, particularly for varsity and contact-sport athletes, to address the unique risks faced by high school athletes. Pre-participation exams and ongoing education should focus on avoiding risky substances and behaviors to foster long-term health and well-being.

### Interrelationships Among Lifestyle Pillars

While each pillar of Lifestyle Medicine offers distinct benefits, the reviewed studies suggest that these domains are interrelated, often influencing one another in ways that amplify both risk and resilience in high school athletes. For example, multiple studies highlight how insufficient sleep contributes to heightened stress and impaired cognitive function. Sufrinko et al demonstrated that athletes receiving ≤5 h of sleep performed significantly worse on verbal memory (F = 11.60, *P* = .001), visual memory (F = 6.57, *P* = .01), and reaction time (F = 5.21, *P* = .02) compared to those receiving ≥9 h, indicating a dose–response effect on neurocognitive performance.^28^ Similarly, inadequate nutrition was frequently linked to injury risk and delayed recovery. Magee et al reported that 52.1% of female high school athletes were at risk for low energy availability (LEA), which is associated with increased rates of stress fractures, menstrual irregularities, and impaired bone development, especially during periods of rapid growth and hormonal fluctuation.^24^ This risk may be further compounded in athletes who also experience sleep deficits, as recovery and tissue repair are dependent on adequate energy intake and rest. There is also a strong connection between stress and social support. Delfin et al found that higher levels of emotional and familial support were negatively correlated with psychological stress (r = −0.386 and r = −0.412, respectively), suggesting that social connection can serve as a protective factor in high-stress sports environments.^32^ Athletes with low perceived support may be more susceptible to the effects of poor sleep and nutritional deficits, further reinforcing the interconnectedness of these lifestyle pillars.

These findings underscore the importance of designing interventions that consider the interplay among lifestyle domains. Addressing only one pillar, such as adding a sleep hygiene program, without acknowledging co-occurring issues like poor dietary intake or lack of social support may limit the overall effectiveness of interventions aimed at optimizing adolescent athletic performance and well-being.

## Discussion

The findings of this review highlight the significant impact that lifestyle factors have on the performance and well-being of high school athletes. While this population faces unique challenges due to their developmental stage, academic pressures, and athletic demands, Lifestyle Medicine’s six pillars, nutrition, sleep, stress management, physical activity, avoidance of risky substances, and social connection, offer a comprehensive framework for optimizing their health and performance.^49^

While the primary focus of this review is on high school athletes, comparing these findings with studies on collegiate and professional athletes offers valuable insights. For example, research on collegiate and professional athletes has consistently demonstrated the importance of proper nutrition for endurance, strength, and recovery.^12,50,51^ Similar outcomes are seen in high school athletes, but the consequences are often more severe due to ongoing growth spurts and developmental needs. Nutritional deficiencies can have more profound effects on adolescent athletes, potentially leading to stunted growth, decreased bone density, and impaired muscle development.^52^ Boisseau et al emphasize that adolescents require higher relative protein intake to support muscle development, while Petrie et al highlight the connection between inadequate calcium intake and long-term skeletal complications in youth athletes.^21,22^ These findings demonstrate the heightened urgency for early nutritional interventions in this population.

### Policy Implications

The results of this review support the need for systemic changes in high school athletic programs. Given the 52.1% prevalence of low energy availability (LEA) in female athletes reported by Magee et al and its association with disordered eating and menstrual irregularities, there is a critical need for school-based nutrition education.^24^ Policies should mandate the integration of comprehensive nutrition programs into athletic departments, with a focus on fueling for performance and menstrual health awareness. In addition, incorporating mental health screenings into routine pre-participation physicals could help detect early signs of stress, burnout, or disordered eating behaviors.^53,54^ Schools should also consider funding sleep hygiene workshops, given the known effects of poor sleep on academic and athletic performance. Such interventions could mirror those used in professional sports, where structured sleep regimens have improved recovery and cognitive function. These policy changes would address not only performance optimization but also long-term health equity.

### Cultural and Socioeconomic Disparities

Several studies reviewed underscore disparities rooted in socioeconomic and cultural contexts, especially among Latino athletes. Manore et al found that lower nutrition knowledge and reduced access to healthy foods significantly impacted dietary behaviors in underserved youth.^23^ Athletes from low-income communities often face limited availability of nutritious meals, lack of safe training environments, and fewer mental health resources, barriers that directly hinder the adoption of lifestyle interventions. Addressing these gaps requires community-based solutions, such as school gardens, culturally relevant dietary education, and subsidized training programs. These findings suggest that one-size-fits-all interventions are insufficient and must be tailored to the unique needs of different cultural groups and geographic regions. Lastly, cultural and regional variations were often overlooked. Social connection, for instance, may play varying roles in different cultural settings, where societal expectations and team dynamics around sports can differ widely. High school athletes from diverse socioeconomic and cultural backgrounds may encounter unique challenges that were not adequately captured in existing studies. Future research should aim to include more diverse populations and explore how cultural and regional factors may influence the impact of lifestyle interventions on athletic outcomes.

### Practical Applications for Coaches, Athletic Trainers, and Healthcare Providers

Based on the findings from this review, several actionable interventions can be implemented in high school sports programs to enhance the well-being and performance of athletes. Coaches and athletic trainers should take a proactive approach by incorporating lifestyle education into their training regimens. For example, they can provide workshops on balanced nutrition and hydration strategies tailored to meet the needs of adolescent athletes. These workshops should emphasize the importance of macronutrient timing, such as carbohydrate loading before games and protein consumption post-training, to optimize energy levels and recovery.^12,55^

In terms of sleep, coaches and trainers can encourage athletes to establish consistent sleep routines and educate them on sleep hygiene practices, such as limiting screen time before bed and creating a sleep-conducive environment.^56,57^ This can be reinforced by implementing technology-free periods during team activities or providing resources like sleep tracking apps to help athletes monitor and improve their sleep habits. Stress management techniques, such as mindfulness exercises, should be incorporated into regular training sessions. Coaches could lead short breathing exercises or mindfulness meditations before practices or competitions, helping athletes develop the tools to manage stress in high-pressure situations. Additionally, creating a team culture that prioritizes mental health, encourages open communication, and provides access to counseling resources can help reduce the stigma associated with seeking help for stress and anxiety. Healthcare providers who work with adolescent athletes also play a crucial role in ensuring that lifestyle factors are assessed during routine checkups. Providers should ask about an athlete’s diet, sleep patterns, and stress levels, offering personalized advice on how to optimize these areas for better performance. They can also collaborate with schools and athletic departments to offer seminars on substance use prevention, nutrition, and mental health.

## Limitations and Future Research

This review encountered several limitations that should be addressed in future research. One significant limitation is the limited availability of research focused specifically on high school athletes. Much of the existing literature examines collegiate and professional athletes, whose physiological, psychological, and social environments differ significantly from those of adolescents. Consequently, findings from studies on older athletes may not fully capture the unique needs and developmental considerations of the high school athlete population. Additionally, this review did not have a registered protocol (e.g., PROSPERO), which may limit the reproducibility and transparency of the review process. Future studies should consider protocol registration to enhance methodological rigor and minimize bias. Additionally, only English-language studies were included, raising the potential for language bias. These exclusions may affect the generalizability of findings across non-English speaking or global populations. The reliance on cross-sectional and self-reported data introduces risks of recall bias and restricts the ability to assess causality or long-term outcomes. Few studies used standardized outcome measures, limiting the comparability of findings. Moreover, potential publication bias may have led to an overrepresentation of studies with positive findings.

Additionally, many of the studies included in this review relied on cross-sectional designs, limiting the ability to assess long-term effects of lifestyle interventions on athletic performance. Future research would benefit from adopting longitudinal designs to better understand how sustained lifestyle interventions impact high school athletes’ performance, injury rates, and psychological well-being over time. Another limitation stems from the reliance on self-reported data, particularly for nutrition, sleep, and stress management practices. Self-report measures are vulnerable to recall bias, which may compromise the accuracy of findings. Future studies should consider incorporating objective measures, such as wearables for sleep tracking and dietary logs verified by nutritionists, to improve data accuracy. Furthermore, a lack of standardization in outcome measures, such as performance metrics, injury rates, and psychological well-being, poses challenges for drawing generalizable conclusions. Future studies should prioritize standardized measurement tools to enhance comparability across research in this area. This review is limited by the uneven distribution of studies across the six pillars of Lifestyle Medicine, with certain areas, such as physical activity, being underrepresented. Additionally, the lack of longitudinal data restricts the ability to evaluate the sustained effects of interventions on adolescent athletes over time. Future research should aim to address these gaps by incorporating more balanced investigations across all pillars and designing longitudinal studies that capture both short- and long-term outcomes.

Future research should aim to include more diverse populations and explore how cultural and regional factors may influence the impact of lifestyle interventions on athletic outcomes. Additionally, researchers should explore the differential impacts of the six lifestyle pillars across various sports, providing insights into which interventions are most beneficial for specific types of performance outcomes. To improve the adoption of healthier lifestyles among high school athletes, targeted policy changes are essential, especially in underserved regions where access to supportive resources may be limited. Equitable access to mental health support, nutritious food options, and safe physical activity spaces should be prioritized to address disparities that can influence an athlete’s ability to implement and sustain healthy lifestyle practices. Policies could include funding for mental health services within schools, partnerships with local food programs to provide access to nutritious meals, and initiatives to improve athletic facilities and physical education programs in underserved areas.

## Conclusion

This review underscores the urgent need to address the lifestyle factors that critically influence the health and performance of high school athletes during a pivotal stage of physical and psychological development. The six pillars of Lifestyle Medicine, nutrition, sleep, stress management, physical activity, avoidance of risky substances, and social connection, are not only performance-enhancing strategies but essential components of adolescent health promotion. With alarming findings such as a 52.1% prevalence of low energy availability (LEA) among female athletes and widespread sleep deficiencies, there is a clear imperative for stakeholders, including schools, coaches, athletic trainers, and policymakers, to take immediate action. Implementing evidence-based interventions such as mandated nutrition education, mental health screenings during pre-participation exams, and structured sleep hygiene programs can create a supportive infrastructure that fosters both athletic success and long-term well-being. Future research must continue to explore and validate such interventions, but the evidence already compels us to act. The time to integrate holistic, developmentally informed practices into high school sports programs is now before preventable health consequences take root in our nation’s youth.
